# Nitric oxide releases Cl^−^ from acidic organelles in retinal amacrine cells

**DOI:** 10.3389/fncel.2015.00213

**Published:** 2015-06-08

**Authors:** Vijai Krishnan, Evanna Gleason

**Affiliations:** Department of Biological Sciences, Louisiana State UniversityBaton Rouge, LA, USA

**Keywords:** cytosolic chloride, amacrine cells, nitric oxide, GABA receptors, synaptic transmission

## Abstract

Determining the factors regulating cytosolic Cl^−^ in neurons is fundamental to our understanding of the function of GABA- and glycinergic synapses. This is because the Cl^−^ distribution across the postsynaptic plasma membrane determines the sign and strength of postsynaptic voltage responses. We have previously demonstrated that nitric oxide (NO) releases Cl^−^ into the cytosol from an internal compartment in both retinal amacrine cells and hippocampal neurons. Furthermore, we have shown that the increase in cytosolic Cl^−^ is dependent upon a decrease in cytosolic pH. Here, our goals were to confirm the compartmental nature of the internal Cl^−^ store and to test the hypothesis that Cl^−^ is being released from acidic organelles (AO) such as the Golgi, endosomes or lysosomes. To achieve this, we made whole cell voltage clamp recordings from cultured chick retinal amacrine cells and used GABA-gated currents to track changes in cytosolic Cl^−^. Our results demonstrate that intact internal proton gradients are required for the NO-dependent release of internal Cl^−^. Furthermore, we demonstrate that increasing the pH of AO leads to release of Cl^−^ into the cytosol. Intriguingly, the elevation of organellar pH results in a reversal in the effects of NO. These results demonstrate that cytosolic Cl^−^ is closely linked to the regulation and maintenance of organellar pH and provide evidence that acidic compartments are the target of NO.

## Introduction

The regulation of cytosolic Cl^−^ plays a key role in determining the inhibitory strength within neuronal circuits. The postsynaptic effect of the neurotransmitters GABA and glycine acting on their ionotropic receptors is dependent upon the distribution of Cl^−^ across the plasma membrane. Thus, regulation of intracellular Cl^−^ levels influences the sign and synaptic strength of GABAergic and glycinergic synapses by influencing the reversal potential of GABA- or glycine-gated synaptic currents. In the retina, GABA and glycinergic amacrine cells are known to generate inhibitory output onto bipolar cells, ganglion cells and other amacrine cells. This signaling is known to be key in regulating the response properties of different classes of retinal ganglion cells (Zhou and Lee, 2008; Masland, 2012; Venkataramani et al., 2014). Therefore, transient and possibly localized modifications in cytosolic Cl^−^ levels have the potential to alter synaptic signaling in the inner retina and ultimately, the output of the retina.

The expression and function of the Na-K-Cl (NKCC) and K-Cl co-transporters (KCC) are well known to adjust cytosolic Cl^−^ during development (Payne et al., 2003) and in some adult neurons (for review, see Kaila et al., 2014). More recently it has been reported that the level of internal and external organic anions may also be a determinant of the distribution of Cl^−^ across the neuronal plasma membrane (Glykys et al., 2014). Almost completely neglected, however, is a consideration of the role of subcellular compartments that harbor Cl^−^ and their ability to release Cl^−^ into the cytosol.

**Figure 1:**
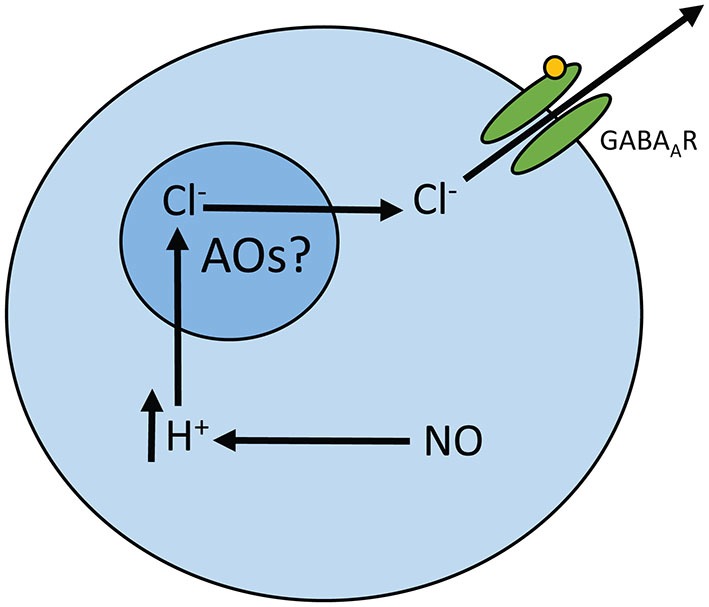
**Model for NO-dependent release of internal Cl^−^**. This model depicts our understanding of the NO-dependent release of Cl^−^ as well as the hypothesis tested in this paper. With NO, a cytosolic acidification occurs that is required for internal Cl^−^ release. The presence of Cl^−^ in the cytosol can be measured as an inward Cl^−^ current due to Cl^−^ leaving open GABAARs. Here, we test the hypothesis that the NO releasable Cl^−^ originates from acidic organelles (AOs).

Previous work from our lab has demonstrated that nitric oxide (NO) can alter the signaling properties of neurons by releasing Cl^−^ from an internal compartment in cultured avian retinal amacrine cells and rat hippocampal neurons (Hoffpauir et al., 2006). All organelles are known to contain some Cl^−^ and express mechanisms to transport Cl^−^ (for review, see Edwards and Kahl, 2010). For example the endoplasmic reticulum and mitochondria contain Cl^−^ and express Cl^−^ transporters but have neutral and basic (matrix) pH, respectively. There is a strong correlation, however, between the level of acidity in organelles and their Cl^−^ content (Sonawane and Verkman, 2003) (for review, see Stauber and Jentsch, 2013). Because of this relationship, we hypothesize the NO releases Cl^−^ from Cl^−^ rich acidic organelles (AO; Figure 1). These compartments include the Golgi, endosomes, synaptic vesicles, and lysosomes.

Here we test the hypothesis that AOs are the source of NO-releasable Cl^−^. To achieve this, we use whole cell voltage clamp recordings on cultured amacrine cells derived from the chicken retina. Chick retinal amacrine cells have been well-characterized and together with our previous related studies provide an ideal model system for further elucidating mechanisms underlying Cl^−^ homeostasis in central neurons. Here, we further investigate the influences of the Cl^−^ store on cytosolic Cl^−^ and confirm that this store is compartmentalized. Additionally, we demonstrate that internal proton gradients are required for the NO-dependent release of internal Cl^−^. Furthermore, we examine the role of compartmental pH and find that it is key in determining cytosolic Cl^−^ levels, both before and after an NO stimulus. Overall, these results support the hypothesis that NO-dependent release of cytosolic Cl^−^ is coming from AOs and link compartmental pH to the function of GABAergic synapses.

## Materials and Methods

### Cell Culture

Primary culture methods have been previously described (Hoffpauir and Gleason, 2002). Our methods were determined to be exempt by the LSU Institutional Animal Care and Use Committee (IACUC). Briefly, retinae from 8 day chick embryos were dissected and separated from the pigment epithelium. Retinal tissue was mechanically dissociated in calcium free Hank’s solution (Life Technologies, Grand Island, NY). The tissue was centrifuged, re-suspended and treated with trypsin (0.125%) for 30 min. Following trituration with DNAase I (Sigma-Aldrich, St. Louis, MO) and centrifugation, cells were re-suspended in Dulbeco’s Modified Eagle’s Medium (DMEM, Life Technologies) supplemented with 5% fetal calf serum (Hyclone, Logan UT) and 1,000 U of penicillin/mL, 100 μg streptomycin/mL and 2 mM glutamine (Life Technologies). Cells were plated on to poly-L-ornithine treated dishes at a density of 2.5 × 10^5^ cells/35 mm dish. One day after plating, DMEM was replaced with Neurobasal, 1% B-27 nutrient medium (Life Technologies) and pen-strep glutamine (Sigma-Aldrich). Cells were fed every other day until the cultures were no longer viable for experimentation (10–12 days). Amacrine cells were identified based on their morphology as previously described (Gleason et al., 1993).

### Electrophysiology

Amacrine cells were used for electrophysiological experiments after 6–12 days in culture. Culture dishes were mounted on the stage of an inverted Olympus IX-70 microscope. A reference Ag/AgCl pellet in 3M KCL was connected to the dish via a 3M KCL and agarose-filled glass bridge. Patch electrodes with tip resistance values of (8–11 MΩ) were pulled from thick-walled borosilicate glass (Sutter instruments, Novato, CA) using a P-97 micropipette puller (Sutter Instruments). Electrophysiology experiments were performed in the whole cell voltage clamp mode using Axopatch 1D-patch clamp (Molecular Devices, Sunnyvale, CA) and Clampex 9.2 software (Molecular Devices). For experiments involving voltage ramps, corrections were made for errors due to junction potential and series resistance.

### Solutions

Unless otherwise specified, reagents were obtained from Sigma-Aldrich For recordings carried out in Cl^−^ free condition, the external solution contained (in mM) Na methanesulfonate 145.0, glucose 5.6 and HEPES 10.0. External solutions were pH adjusted to 7.4 with NaOH. The internal zero Cl^−^ solution consisted of Cs methanesulfonate 145.0 and HEPES 10.0. Although these solutions lack some of the normal ions, the results of our GABA pulse experiments and the ramp experiments are consistent indicating that the zero Cl^−^ solutions are not problematic. As an additional control we have done zero Cl^−^ GABA pulse experiments with normal Ca^2+^ in the bath and find no differences in responses. Voltage ramp experiments were performed using TEA-Cl^−^ external contained the following ingredients (in mM): NaCl 116.7, KCl 5.3, TEA-Cl 20.0, CaCl_2_ 3.0, MgCl_2_ 410 μM, HEPES 10.0, and glucose 5.6. The TEA-Cl external solution was supplemented with 300 nM TTX (Alomone Labs, Jerusalem, Israel) and 50 μM LaCl_3_ to block voltage-gated sodium and calcium channels, respectively. The internal pipette solution for voltage ramp experiments contained in mM: cesium acetate 100.0, CsCl 10.0, CaCl2 0.1, MgCl2 2.0, HEPES 10.0 and EGTA 1.1 along with the ATP regeneration system. For experiments involving methylamine hydrochloride, methylamine replaced CsCl to maintain E_Cl^−^_. The internal recording solutions were pH adjusted to 7.4 with CsOH. Both pipette solutions were supplemented with an ATP regeneration system containing: 50U/ml creatine phosphokinase, 20 mM creatine phosphate, 1 mM ATP disodium, 3 mM ATP dipotassium and 2 mM GTP disodium. Methylamine hydrochloride (MA, 10 mM), chloroquine (CQ, 100 μM) were added to pipet solutions. Carbonyl cyanide 4-(trifluoromethoxy) phenylhydrazone (FCCP 1.0 μM) and bafilomycin (1.0 μM) were added to the bath. External solutions were controlled by a ValveLink pinch valve gravity perfusion system (Automate Scientific, Berkeley, CA). For whole cell voltage clamp experiments, solution changes were achieved with a computer-controlled automated SF-77B perfusion fast stepper (Warner Instruments, Hamden, CT). As a control, cells were routinely exposed to NO-free, low pH solution (McMains and Gleason, 2011) and none of the results reported here were reproduced by this treatment. Estimations of cytosolic Cl^−^ content were made without considering the contribution of acetate which for these GABA_A_ receptors (GABA_A_Rs) seems to be small because we observe that under normal Cl^−^ and control conditions, E_Cl^−^_ and E_GABA_ are within a few millivolts of each other.

### NO Solutions

NO was prepared by bubbling Fisher ultra-distilled pure water with argon for 15 min followed by bubbling with pure NO gas for 15 min. The NO was then sealed and protected from light and stored at room temperature until use. NO (30–50 μL) was injected into the perfusion line and has been previously estimated to reach the recorded cell in ~3 s and to remain for ~3–5 s (Hoffpauir et al., 2006). Equal volumes of NO-free distilled water were routinely injected and none of the results reported here were replicated by this manipulation. The NO donor 1-Hydroxy-2-oxo-3-(3-aminopropyl)-3-isopropyl-1-triazene (NOC-5) was obtained from Dojindo Molecular Technologies (Rockville, Maryland) and stored at −20°C. NOC-5 solutions were prepared in zero Cl^−^ external solution just prior to use.

### Data Analysis

Decay indices (Figure 3) were calculated by the following formulae: DI = 1 − (amp P5/amp P1) or DI = 1 − (amp P2/amp P1) with “amp” indicating GABA-gated current amplitude. Data were analyzed using Origin 8.0 (OriginLab, Northampton, MA) analysis software and data are presented as means ± SD. Data were generally evaluated using the paired and unpaired student’s *t*-test as appropriate. Levels of significance are denoted by *p < 0.05, **p < 0.01, ***p < 0.001, ****p < 0.0001.

## Results

### The Cl^−^ Store is Sequestered

**Figure 2:**
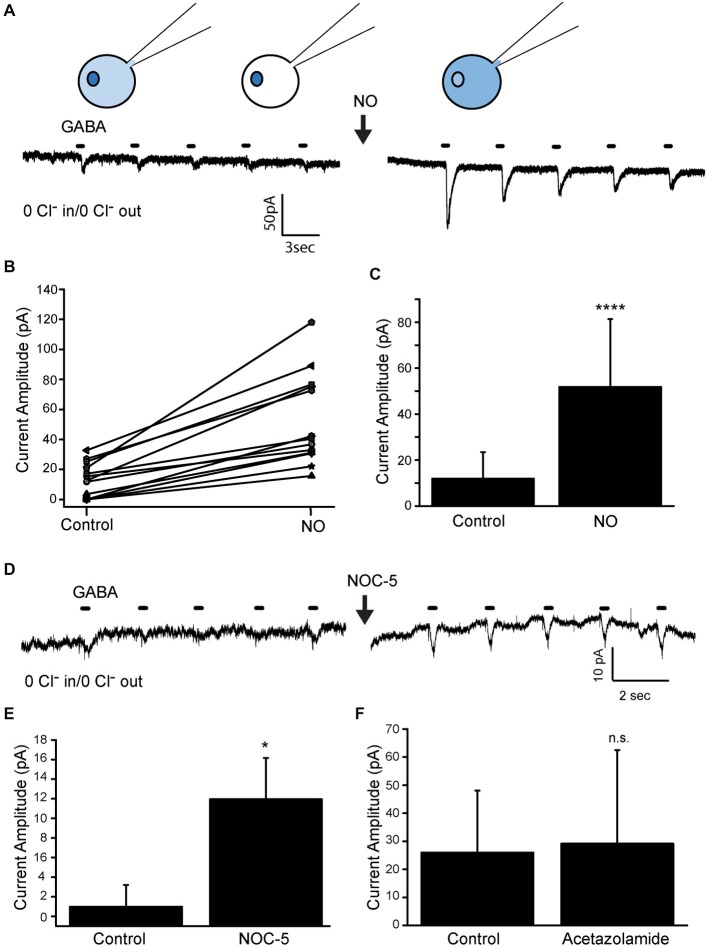
**The NO-dependent Cl^−^ store is sequestered. (A)**, Representative traces from an amacrine cell recorded in the whole-cell voltage clamp configuration with Cl^−^ free pipette and external solutions. The cell was held at −70 mV and pulses of GABA (20 μM, 400 ms) were applied. Injection of NO temporarily increased the amplitude of the GABA-gated currents indicating a release of Cl^−^ into the cytosol. Cartoons at the top depict the Cl^−^ distribution at the point in time being sampled directly below. The darker color represents more Cl^−^. **(B)**, Current amplitude data are plotted for each cell recorded. Data were collected from the response to the first GABA pulse and from the response to the 6th GABA pulse delivered just after NO. **(C)**, Mean current amplitude of the data shown in **(B)**. **(D,E)**, Data from an experiment like the one shown in **(A)** but here the NO donor NOC-5 (500 μM) is used rather than the NO-bubbled solution. The NO donor also causes a significant increase in the amplitude in the NO-dependent GABA-gated currents. **(F)**, Mean NO-dependent current amplitude of GABA-gated currents recorded before and after the addition of acetazolamide (400 μm). **** denotes *p* < 0.0001.

Our previous work has supported the regulated communication between three separate Cl^−^ containing compartments: an intracellular compartment, the cytosolic compartment and the extracellular compartment. The intracellular compartment is envisioned to be a membrane bound compartment but an alternative hypothesis is that the NO-releasable Cl^−^ is not sequestered but is instead bound up to proteins in the cytosol (Fiedler et al., 2000; Hörnberg et al., 2005; Zhou et al., 2010). To determine whether the store is actually contained within an intracellular membrane-bound compartment, we examined the GABA-gated currents in the absence of both external and recording pipet Cl^−^. GABA-gated current in cultured amacrine cells are known to be entirely due to the activation of GABA_A_Rs. The assumption in this experiment is that the Cl^−^-free pipet solution can dialyze the cytosol and will eventually wash out both cytosolic and protein-bound Cl^−^. Cells recorded in the whole cell recording configuration (ruptured patch) were held at −70 mV, and five, 400 ms pulses of GABA (20 μM) were delivered. Inward GABA-gated currents were often observed in response to the first few GABA applications, but when observed, these currents typically dissipated as Cl^−^ washed out of the cytosol (see below). In all cells tested (*n* = 14), after GABA-gated currents were no longer visible (see 5th GABA application before NO, Figure 2A), injection of NO produced inward GABA-gated currents consistent with Cl^−^ being released from an internal membrane bound compartment and exiting the cell via GABA_A_Rs (Figures 2B,C, mean current amplitude pre NO 11.9 pA SD (11.6 pA); post NO 51.8 pA SD (29.5 pA); *n* = 14; *p* < 0.0001). To rule out any involvement of a GABA transport current, muscimol was substituted for GABA. Muscimol activates GABA_A_Rs but does not activate the GABA transporter. With muscimol as the agonist, the NO-dependent appearance of inward current was still observed (mean current amplitude pre NO 3.0 pA SD (3.3 pA); post NO 66.3 pA SD (26.9 pA); *n* = 6, not shown). To confirm the involvement of NO, this same experiment was done using the NO donor NOC-5 (500 μm, Figures 2D,E). NOC-5 also increased the amplitude of the GABA-gated currents (mean current amplitude control 1.0 pA SD (2.2 pA); NOC-5 12.0 pA SD (4.2 pA); *n* = 5; *p* = 0.01) further supporting an NO-dependent release of Cl^−^ from an internal store.

Because both Cl^−^ and bicarbonate (HCO_3^−^_) permeate GABA_A_ receptors (Bormann et al., 1987), it is possible that an increase in intracellular HCO_3^−^_ could contribute to the NO-dependent increase in GABA-gated current amplitude. Although this is unlikely because these experiments have been conducted in HEPES-buffered solutions, the cells are still producing metabolic CO_2_ and it is possible that NO stimulates an endogenous carbonic anhydrase (CA) in amacrine cells. Expression of CA2 has been demonstrated for a subset of amacrine cells in the developing chick retina but is mostly confined to Müller glia in the adult chicken retina (Linser and Moscona, 1984). To rule out the unlikely participation of CA-generated HCO_3^−^_ in the NO-dependent increase in GABA-gated current amplitude, the CA inhibitor acetazolamide was used (Figure 2F). The same protocol was followed as for the experiments shown in Figures 2A,D. No effect of acetazolamide on the NO-dependent GABA-gated currents were observed (mean current amplitude control 26.0 pA SD (22.0 pA); acetazolamide 29.2 pA SD (33.5 pA); *n* = 6; *p* = 0.6) providing evidence that CA-dependent HCO_3^−^_ is not involved in the reappearance of GABA-gated current after NO.

### Influence of the Patch Pipette on Cytosolic Cl^−^

**Figure 3:**
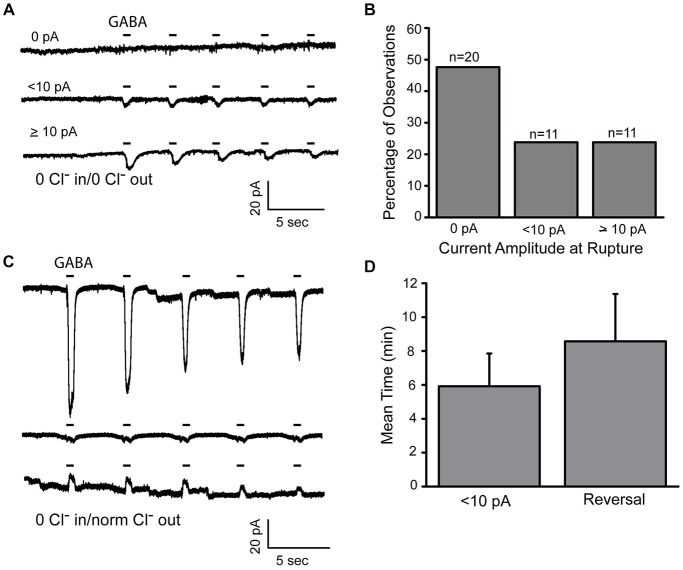
**Cytosolic Cl^−^ is incompletely controlled by the patch pipet. (A)**, Representative recordings from three different amacrine cells made within 5 s of membrane rupture. Cells were recorded with zero Cl^−^ pipet and extracellular solutions and were held at −70 mv. GABA (20 μM) was applied in 400 ms pulses. **(B)**, Forty-two cells recorded under the conditions in **(A)** are categorized by the initial amplitude of their GABA-gated currents. **(C)**, Representative recordings from a single amacrine cell, 5 s (top), 6 min (middle) and 14 min (bottom) after membrane rupture. Recording conditions are the same as in **(A)** except that the extracellular solution contains normal Cl^−^ concentration. **(D)**, Data are plotted from cells recorded under the conditions in **(C)**.

As indicated above, there was often evidence for residual Cl^−^ in the cytosol even with a Cl^−^ free internal solution. To more fully define the influence of the patch pipet in these experiments, we recorded from a population of cells and looked at the fraction of cells that retained inward Cl^−^ currents in recordings obtained immediately after patch rupture (Figures 3A,B). We observed that about half the cells showed residual inward GABA-gated currents (0 pA 47.6%; <10 pA 23.7% >10 pA 23.7%, *n* = 42). The time required to dialyze cytosolic Cl^−^ with repeated delivery of the 5 GABA pulse protocol in those cells that demonstrated inward currents was also estimated and this time period ranged from about 10–60 s (mean = 18.2 s SD (14.6 s)). Although the time frame could vary, elimination of the GABA-gated current was always achieved. In these experiments, we are assuming that loss of GABA-gated current indicates the absence of cytosolic Cl^−^. It is possible, however, that the loss of the current is due to reduced activity of the receptors themselves. To address this possibility, we recorded from amacrine cells in normal external Cl^−^ but with zero Cl^−^ pipet solution. Under these conditions, loss of internal Cl^−^ should lead to an actual reversal of the GABA-gated current from inward to outward. With normal extracellular Cl^−^, we observed that the time course of depletion was prolonged, presumably due to Cl^−^ import mechanisms operating at the plasma membrane opposing the influence of the zero Cl^−^ pipet. Nonetheless, over the time course of minutes (<10 pA mean 5.9 min SD (5.5 min); reversal mean 8.6 min SD (6.8 min)), the GABA-gated current reversed (Figures 3C,D) indicating that the GABA_A_ receptors were still functional.

**Figure 4:**
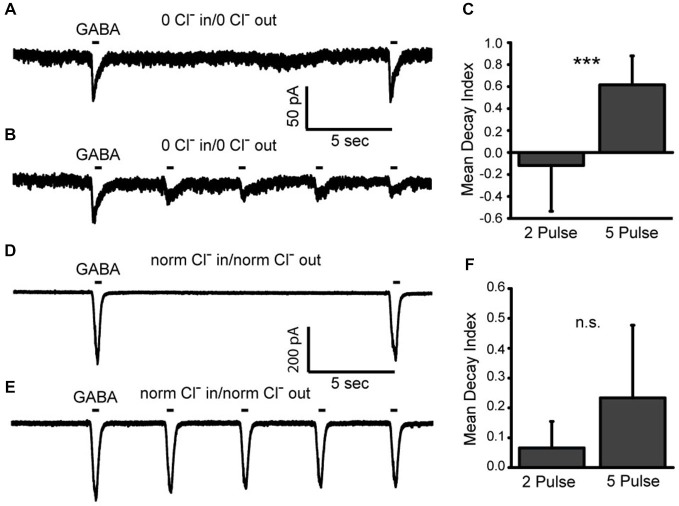
**Cl^−^ escape via GABA_A_ receptors can be offset by extracellular Cl^−^. (A,B)**, Whole cell recordings made from two different amacrine cells immediately after membrane rupture. In the first, two 400 ms pulses of GABA are applied and in the second, five are applied. Recordings were made under zero Cl^−^ conditions. **(C)**, Decay indices were calculated as indicated in the Methods. These data indicate that the decay in the amplitude of GABA-gated currents is accelerated when the receptors are activated more frequently. **(D,E)**, Data from two different amacrine cells recorded as in **(A)** but in the presence of normal intra and extracellular Cl^−^ concentrations. **(F)**, Decay indices are plotted for cells recorded under the conditions in **(C)** and no significant difference in current amplitude is observed. *** denotes *p* < 0.001.

It is expected that the flux of Cl^−^ through the GABA_A_ receptors themselves contributed to the cytosolic Cl^−^ depletion that we observe in Figure 3. To evaluate this, we examined the effect that varying the number of GABA pulses delivered had on the current amplitude (Figure 4). We compared the GABA-gated current amplitudes elicited during the first recording after patch rupture. Some cells were tested with the usual 5 GABA pulse protocol while others were tested with a 2 GABA pulse protocol (delivered over the same time frame). A decay index (DI) was calculated (see Methods Section) for each recording. Recordings were made either in zero Cl^−^ solutions inside and out (Figures 4A,C) or with normal internal Cl^−^ and normal external Cl^−^ solutions (Figures 4D,E). In complete zero Cl^−^ solutions, the 5 pulse protocol showed a significantly larger DI indicating that, as expected, the increased opening of GABA_A_Rs contributes to the depletion of cytosolic Cl^−^ (Figures 4A,B; mean 2 pulse DI −0.12 SD (0.42); *n* = 9; mean 5 pulse DI 0.62 SD (0.26); *n* = 8; *p* = 0.007) Interestingly, there was no significant difference in the DI for the cells recorded in normal Cl^−^ (Figures 4D–F; mean 2 pulse DI 0.07 SD 0.09; *n* = 6; mean 5 pulse DI 0.23 SD (0.2); *n* = 7; *p* = 0.1). This result suggests that Cl^−^ import mechanisms are able to minimize the effect of the loss of cytosolic Cl^−^ through GABA_A_Rs under these conditions. Together, these results demonstrate that although pipet Cl^−^ eventually dominates over cytosolic Cl^−^, this effect is not immediate, is variable from cell to cell and is affected by extracellular Cl^−.^. Most importantly, the zero Cl^−^ experimental design allows us to isolate the internal store unequivocally.

### Disruption of Compartmental Proton Gradient Alters the NO-Dependent Cl^−^ Release

**Figure 5:**
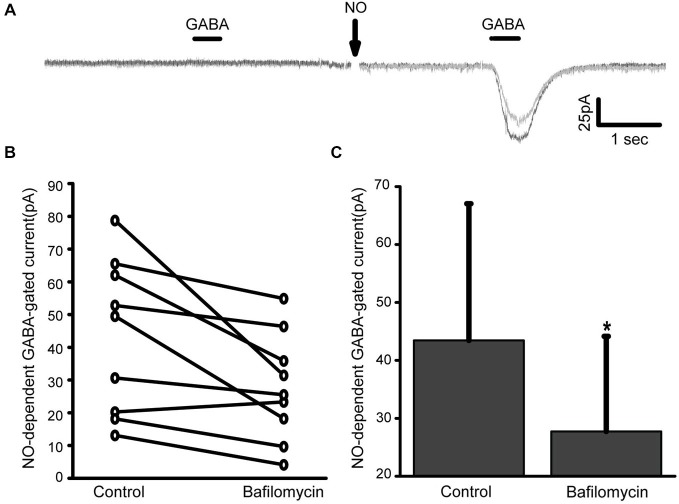
**Reducing AO proton gradients inhibits the NO-dependent release of Cl^−^. (A)**, Data from a representative amacrine cell before and after addition of 1 mM bafilomycin under zero Cl^−^ conditions. On the left, GABA produces no current indicating that the cytosol is Cl^−^ free. After NO GABA elicits an inward current due to Cl^−^ moving from the cytosol out across the plasma membrane. After bafilomycin (gray trace), the NO-dependent GABA-gated current is smaller indicating a reduction in the release of internal Cl^−^. **(B)**, NO-dependent GABA-gated current amplitude is plotted for each cell tested both before and after exposure to bafilomycin. **(C)**, Mean GABA-gated current amplitude is significantly smaller in bafilomycin. * denotes *p* < 0.05.

AOs are known to contain millimolar concentrations of Cl^−^ (Sonawane and Verkman, 2003) and are therefore strong candidates for harboring NO-releasable Cl^−^. These organelles also express the V-type proton pump and the activity of this active transporter generates a large proton gradient. If AOs harbor NO-releasable Cl^−^, then it is possible that the proton gradient is linked to the NO-dependent export of Cl^−^. We have previously shown that pre-incubation with the V-type ATPase inhibitor bafilomycin produces a small but significant inhibition in the NO-dependent positive shift in the reversal potential (E_rev_) under normal Cl^−^ conditions (McMains and Gleason, 2011). Here, we examine the effects of bafilomycin applied acutely under zero Cl^−^ conditions to test the hypothesis that there is a relationship between the proton gradient across AO membranes and the level of NO-dependent Cl^−^ release. The NO-dependent increase in GABA-gated current amplitude was examined for amacrine cells held at −70 mV both before and after exposure to bafilomycin (1.0 μM, Figure 5A). We observed that the NO-dependent GABA-gated currents were usually smaller than control (Figures 5B,C) and this was independent of the order of control and bafilomycin treatments. These results suggest that a reduction in the proton gradient across AO membranes impairs the ability of NO to release Cl^−^.

Bafilomycin blocks the V-type ATPase and upsets the balance between inward proton pumping and outward proton leak (Fuchs et al., 1989; Rybak et al., 1997; Wu et al., 2000; Grabe and Oster, 2001). The effect of brief (~30 s) bafilomycin exposure as we used here, depends upon the amplitude of the proton leak. An alternative approach is to induce a proton leak with an ionophore such as carbonyl cyanide 4-(trifluoromethoxy) phenylhydrazone (FCCP). FCCP is typically used to disrupt the mitochondrial proton gradient where its effect will be to allow protons to move down their electrochemical gradient across the inner mitochondrial membrane and into the mitochondrial matrix. FCCP will also disrupt the AO proton gradients (Forgac et al., 1983) and allow protons to move from AOs into the cytosol.

**Figure 6:**
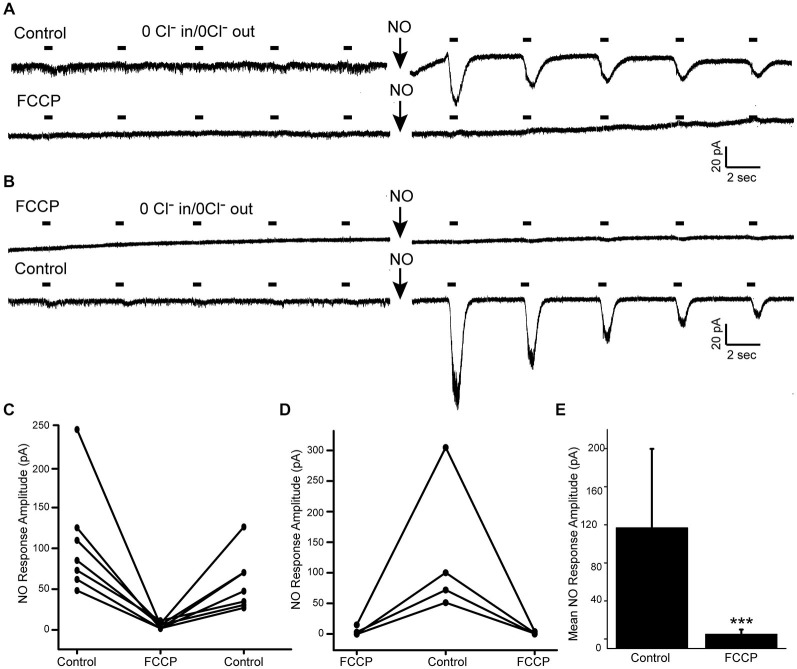
**Internal proton gradients are required for the NO-dependent release of Cl^−^. (A)**, Whole cell recording of an amacrine cell held at −70 mV and exposed to 5 pulses of GABA under Cl^−^ free conditions (timing of pulses indicated by horizontal bars). Control record show some residual GABA-gated currents that diminish over the 5 GABA pulses. After NO, the GABA-gated current amplitude is enhanced indicating and increase in cytosolic Cl^−^. **(A)** (bottom) after FCCP, GABA-gated currents are negligible and after NO, the increase in the GABA-gated current amplitude is not observed. **(B)**, A recording from a different cell where FCCP is applied first. No GABA-gated currents are observed either before or after NO. **(B)** (bottom), A recording made from the same cell after washout of FCCP. NO elicits an increase in the GABA-gated current demonstrating a NO-releasable Cl^−^ store in this cell. **(C,D)**, GABA-gated current amplitude data are plotted for each cell recorded. Cells are sorted according to the order of FCCP application (after control, **(C)**; before control, **(D)**). Data plotted are the GABA-gated current amplitudes recorded just after NO application. **(E)**, Combined mean current amplitude data are plotted to compare control to FCCP. *** denotes *p* < 0.001.

The effects of FCCP were tested in Cl^−^-free GABA pulse experiments. Cells were first shown to have NO-dependent Cl^−^ release by demonstrating the appearance of GABA-gated currents after NO. After FCCP, no NO-dependent GABA-gated currents were detected (Figures 6A,C). If FCCP had caused an emptying of AOs Cl^−^ content, we would expect to see evidence of cytosolic Cl^−^ as inward GABA-gated currents both before and after NO but this was never observed. However, to test this explicitly, some cells were first exposed to FCCP and tested with NO (Figures 6B,D). In these experiments FCCP had the same inhibitory effect on the NO-dependent Cl^−^ release as in the experiment depicted in Figure 6A. FCCP was washed out then the experiment was immediately repeated under control conditions. In all cases, an NO-dependent release of Cl^−^ was observed indicating the Cl^−^ content was preserved during the brief period of the FCCP treatment (~1–2 min). As an additional control, GABA-gated currents recorded under normal Cl^−^ conditions and in the absence of NO were recorded in the presence of FCCP and we found no evidence that FCCP has any direct effect on these receptors. Overall, FCCP had a dramatic inhibitory effect on the ability of NO to release internal Cl^−^ (Figure 6E, mean NO-dependent current amplitude control 116.9 pA SD (82.8); FCCP 5.1 pA SD (4.9), *n* = 11, *p* = 0.001). These results suggests that in the absence of proton gradient, the mechanism underlying the NO-dependent release of internal Cl^−^ is disabled.

### Increasing Compartmental pH Releases Cl^−^ into the Cytosol

Both bafilomycin and FCCP disrupt the normal AO proton gradient which disrupts both the AO membrane potential and increases luminal pH. In order to dissect the role of AO membrane potential from pH, we have tested the effects of lysosomotropic weak bases methylamine (MA) and CQ. These compounds have been demonstrated to alkalinize compartments including lysosomes (Ohkuma and Poole, 1978; Poole and Ohkuma, 1981) and endosomes, including synaptic vesicles (Cousin and Nicholls, 1997; Abreu et al., 2008). When applied via the patch pipet, these compounds will deprotonate in the cytosol then diffuse across organellar membranes. The deprotonated weak base will become trapped when they re-protonate in the acidic environment of the AOs. The dominant effect of these compounds is to increase the pH of acidic compartments without affecting the AO membrane potential (Cousin and Nicholls, 1997). Deprotonation in the cytosol will likely acidify the cytosol to some degree but this effect will be at least partially offset by the activity of the plasma membrane Na/H exchanger (NHE; McMains and Gleason, 2011) and the 10 mM HEPES in the pipet.

**Figure 7:**
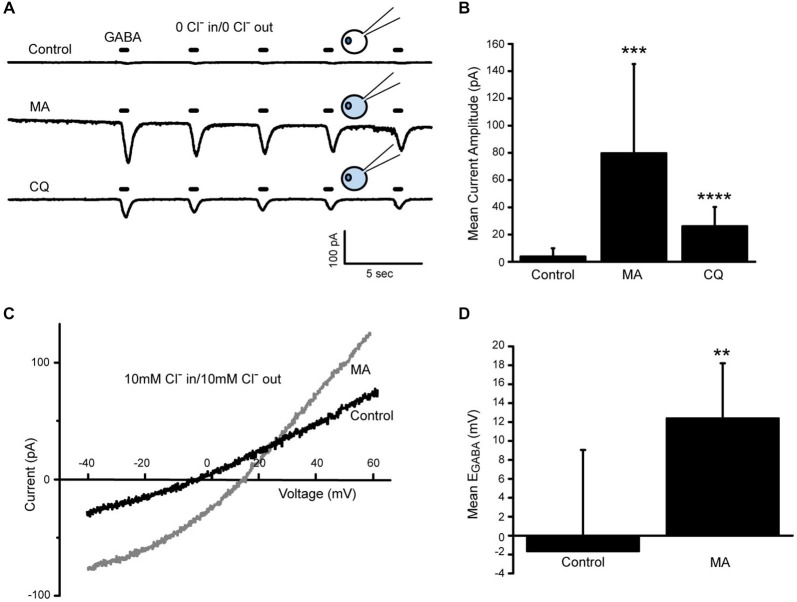
**Increasing compartmental pH releases Cl^−^ into the cytosol. (A)**, Representative traces from three different amacrine cells recorded under zero Cl^−^ conditions. Cells were held at −70 mV. MA and CQ labeled traces are from cells that were recorded with pipet solutions containing these compounds. Traces shown were the first collected after membrane rupture. Cartoons depict cytosolic Cl^−^ levels under the three conditions. **(B)**, Mean amplitudes of the first response to GABA (20 μm, 400 ms) are plotted for each of the three conditions. Both MA and CQ significantly increased the GABA-gated current amplitude over control suggesting higher internal Cl^−^ concentration than control. **(C)**, Leak-subtracted GABA-gated currents recorded from two different amacrine cells in 10 mM Cl^−^ internal and external solutions. Currents were elicited by ramping the voltage from –90 mV to +50 mV in the presence of GABA (20 μM). Recordings were made just after membrane rupture. The control cell has a GABA-gated current reversal potential near the predicted equilibrium potential for Cl^−^ of 0 mV whereas the MA-containing cell’s GABA-gated current reverses at around +15 mV. **(D)**, Cells recorded with MA had significantly more positive reversal potentials than control. ** denotes *p* < 0.01, *** denotes *p* < 0.001, **** denotes *p* < 0.0001.

Either 10 mM MA or 100 μm CQ was included in the pipette solution which was otherwise Cl^−^ free. The external solution was also zero Cl^−^. Recordings were begun about a minute after plasma membrane rupture to allow time for the reagents to gain access to the cell. Amacrine cells were held at −70 mV and exposed to GABA pulses as in previous experiments. In control conditions, the GABA-gated currents were small and often negligible by the fifth GABA pulse. With MA internal, the pre-NO GABA-gated currents were always substantial and did not wash out over the first five GABA pulses (Figures 7A,B; mean current amplitude control 3.9 pA SD (6.1 pA); MA 79.8 pA SD (65.4 pA); *n* = 17; *p* = 0.0003). The larger currents could be entirely due to the 10 mM Cl^−^ in the MA pipet solution (methylamine hydrochloride) or could be due to methylamine somehow stimulating release of Cl^−^ from the internal store. If methylamine was causing release of Cl^−^ from stores because of its ability to buffer protons in AOs, then we would expect to see a similar enhancement of GABA-gated current amplitude with CQ in the pipet.

Confirming this expectation, larger than control GABA-gated currents were consistently observed with CQ in the pipet (Figure 7A, bottom trace). Although CQ had a smaller effect on the pre-NO GABA-gated current amplitude than MA, the difference from control was still strongly statistically significant (Figure 7B; mean current amplitude CQ 26 pA SD (14.2 pA), *n* = 8, *p* < 0.0001). These data suggest that raising the pH of internal compartments releases Cl^−^ into the cytosol in the absence of an NO stimulus. To confirm this and to account for the 10 mM internal Cl^−^ that comes along with the methylamine, the reversal potential of the GABA-gated current (E_GABA_) was determined by applying voltage ramps during application of GABA. These experiments were done in 10 mM external Cl^−^ and either 10 mM CsCl internal solution or 10 mM methylamine HCl internal solution with the predicted E_Cl^−^_ being 0 mV for both combinations. The reversal potential of the GABA-gated current 1–2 min after plasma membrane rupture was determined and these measurements indicated that while control cell currents reversed near 0 mV, the cells containing MA had significantly more positive reversal potentials even though both pairs of solutions had equivalent E_Cl^−^_values (Figures 7C,D; mean E_GABA_ control −1.7mV SD (10.7mV), *n* = 6). The mean E_GABA_ in MA was about +12 mV (MA 12.4 mV SD (5.8 mV), *n* = 9, *p* = 0.006) which would correspond to an additional 6 mM cytosolic Cl^−^.

### Increasing AO pH Reverses the Effect of NO on Cytosolic Cl^−^

**Figure 8:**
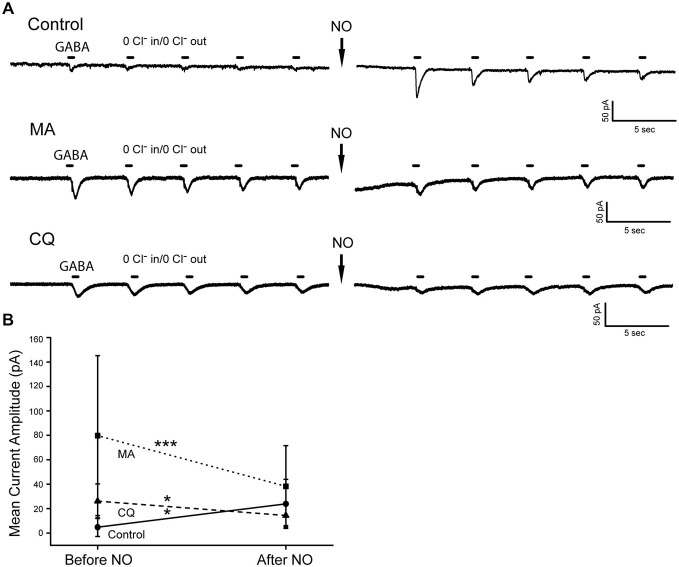
**Increasing compartmental pH eliminates the NO-dependent release of Cl^−^ under zero Cl^−^ conditions. (A)**, Representative recordings from three different amacrine cells before and after addition of NO. The control cell starts out with a small inward GABA-gated current that diminishes over the 5 GABA pulses. After NO, the current is enhanced. In contrast, MA and CQ containing cells had larger GABA-gated currents before NO. After NO, the currents were not enhanced but rather significantly reduced in amplitude. **(B)** Mean current amplitudes are plotted before and after exposure to NO for control, MA and CQ.

If raising the pH in AOs causes Cl^−^ release, how does compartmental pH affect the ability of NO to release Cl^−^ from internal stores? GABA pulse experiments were conducted in zero Cl^−^ solutions to address this question. Control cells initially had typically small GABA-gated currents. After NO, control cells showed the expected increase in the GABA-gated current amplitude indicating an increase in cytosolic Cl^−^ (mean current amplitude control pre-NO 4.8 pA SD (7.6 pA); post-NO 23.9 pA SD (20.1 pA) *n* = 9, *p* = 0.02). As expected, the amplitude of the pre-NO GABA-gated current was larger with MA and CQ internal. However, in cells with MA and CQ, no NO-dependent increase in the GABA-gated current amplitude was observed (Figures 8A,B; MA pre-NO 79.7 pA SD (65.4 pA) post-NO 38.2 pA SD (33.3 pA), *n* = 17; CQ pre-NO 26.1 pA SD (14.2 pA); post-NO 14.4 pA SD (8.3 pA), *n* = 8). In fact, the post-NO GABA-gated currents were significantly reduced rather than enhanced) MA *p* = 0.001; CQ *p* = 0.02) raising the possibility that under these conditions, NO reversed the movement of Cl^−^ between cytosol and AOs or generated export across the plasma membrane.

**Figure 9:**
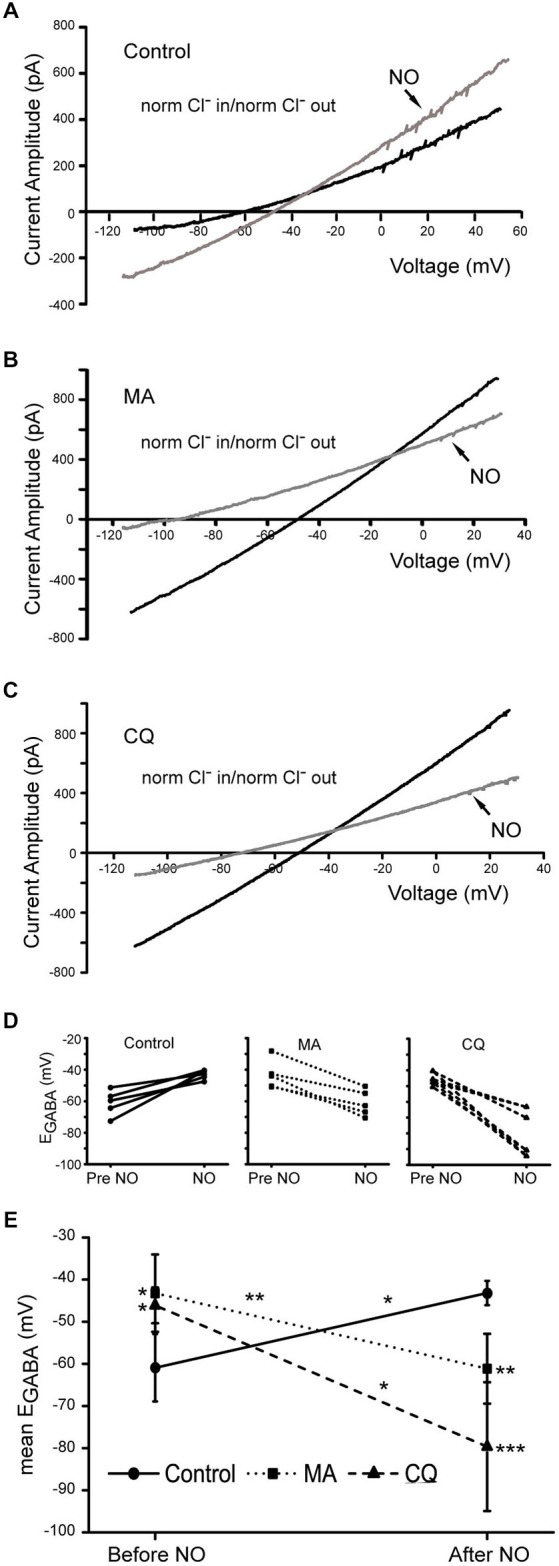
**In normal Cl^−^, increasing compartmental pH promotes NO-dependent Cl^−^ efflux from the cytosol**. GABA-gated currents in response to voltage ramps were recorded in normal internal and external solutions. **(A)**, A recording from a control cell is shown with about a 10 mV positive shift in reversal potential in response to NO. **(B,C)**, GABA-gated currents in cells containing MA or CQ, respectively. In both cases the reversal potentials of the currents before NO are more positive than the calculated E_Cl^−^_, −59 mV. After NO, the reversal potential of the GABA-gated current shifts in the negative direction indicating a decrease in cytosolic Cl^−^. **(D)** NO-dependent shifts in E_GABA_ are shown for individual cells. **(E)**, Data from experiments like A–C are quantified and show that GABA-gated current reversal potentials before NO are significantly more positive with MA and CQ than in control. NO elicits significant shifts in reversal potentials under all three conditions and the post-NO reversal potentials were significantly more negative than control with MA and CQ. Asterisks to the left (pre-NO) and right (post-NO) of the data points indicate significance of differences from control.

To clarify whether the NO response was inhibited or actually reversed, reversal potentials for the GABA-gated currents were measured. These experiments were done in standard Cl^−^ containing solution with Cl^−^_out_ 148.8 mM and Cl^−^_in_ 14.2 mM (E_Cl^−^_ = −59 mV) with MA and CQ added to the internal solution. As expected under control conditions, NO caused a positive shift in E_GABA_ consistent with a release of Cl^−^ into the cytosol (Figure 9A; mean E_GABA_ pre- NO −60.9 mV SD (8.0 mV); post NO −43.2 mV SD (2.9 mV), *n* = 5, *p* = 0.01). These mean values correspond to 14 mM Cl^−^ before NO and 25 mM Cl^−^ after NO (see Methods Section). In this control cell, the NO-dependent GABA-gated current enhancement that we have previously reported was also observed (Hoffpauir et al., 2006). With either MA or CQ internal solution, the E_GABA_ started out significantly more positive than control (Figures 9B–E, MA −43.2 mV SD (9.2 mV), *n* = 5, *p* = 0.01; CQ −46.1 mV SD (4.2 mV), *n* = 4, *p* = 0.04). These E_GABA_ values would correspond to the following cytosolic Cl^−^ values: MA 25 mM and CQ 23 mM. After NO, E_GABA_ shifted in the negative direction indicating an NO-dependent depletion of cytosolic Cl^−^ (Figures 9B–E; MA −61.2 mV SD (8.3 mV); CQ −79.6 SD (15.3mV)). Post-NO values for E_GABA_ were significantly more negative than pre NO values (MA *p* = 0.002; CQ *p* = 0.001). Mean post-NO E_GABA_ values lead to an estimation of 14 mm Cl^−^ in MA and 6 mM Cl^−^ in CQ for cytosolic Cl^−^. We also consistently observed that in both MA and CQ, the GABA-gated ramp current was reduced in amplitude after NO. Presumably, this is a separate effect on the GABA_A_ receptors that might be due to the altered pH environment when NO is co-applied with MA or CQ and could contribute to the post NO reduction in current amplitude we observe in the GABA pulse experiments. Nonetheless, the data in Figure 9 confirm that the reversal potential of the GABA-gated current shifts in the negative direction after NO indicating an NO-dependent reduction in cytosolic Cl^−^.

## Discussion

These results demonstrate that cytosolic Cl^−^ is tightly coupled to the physiological environment of internal stores. Here, we show that a compartmentalized Cl^−^ store releases Cl^−^ into the cytosol after exposure to NO. Reduction of AO proton gradients by acute inhibition of the V-type ATPase limited the NO-dependent release of internal Cl^−^ and more severe disruption of the proton gradient with the protonophore FCCP completely blocked the NO-dependent release of internal Cl^−^. Disruption of compartmental pH with lysosomotropic bases demonstrates two things. First, an increase in compartmental pH without alteration of the AO Vm, allows Cl^−^ to escape into the cytosol. Second, under this same condition, the effect of NO is to promote Cl^−^ uptake or removal rather than release.

### Detecting Cl^−^ Changes in the Ruptured-Patch Configuration

In order to track Cl^−^ release from internal stores specifically, it was important to study the Cl^−^ store in isolation and for this reason, we used ruptured patch in conjunction with zero Cl^−^ solutions. Although our methods in this work were a compromise between isolation of the Cl^−^ store and disruption of the cytosol, it is important to recognize that we have demonstrated in previous work that the NO-dependent release of internal Cl^−^ and its dependance on cytosolic pH are the same in both ruptured patch and gramicidin whole cell recordings (Hoffpauir et al., 2006; McMains and Gleason, 2011).

Although the patch pipet is generally considered to rapidly dialyze the interior of the cell, standing Cl^−^ gradients have been detected in ruptured patch recordings of Clomeleon-expressing hippocampal neurons (Kuner and Augustine, 2000). Our data demonstrate that the washout of Cl^−^ is highly variable from cell to cell, possibly due to differences in cellular architecture. Furthermore, we could readily detect alterations in cytosolic Cl^−^ levels in response to elevated compartmental pH both before and after addition of NO. Although our recording configuration may have minimized changes in cytosolic Cl^−^ or made them more transient, significant changes in Cl^−^ concentration were clearly detectable.

### Interactions Between Cl^−^ and pH

AO Cl^−^ import has been thought to function primarily to provide counter ions to minimize the positive membrane potentials generated by the acidifying activity of the V-type proton pump. However, it has also become clear that endosomal Cl^−^ transport also plays a key role in endocytosis (Piwon et al., 2000; Wang et al., 2000) as well as other aspects of membrane trafficking (for review, see Stauber and Jentsch, 2013). Cl^−^ and pH are also linked by the activity of Cl/ HCO_3_ anion exchangers (AE3) and the Na/Cl/HCO_3_ (NCBE) transporters at the plasma membrane. Interestingly, knockout of AE3 produces increased susceptibility to seizure activity (Hentschke et al., 2006) and knockout of NCBE exhibit decreased excitability and a higher threshold for seizure activity (Jacobs et al., 2008). Both of these defects are consistent with alterations in cytosolic Cl^−^ and/or HCO_3^−^_ that could alter GABA_A_ receptor-mediated inhibition. In the retina, knockout of NCBE led to alterations in the normal response properties of the electro-retinogram (Hilgen et al., 2012). Additionally, a recent report demonstrates that Cl^−^ ions can bind and regulate the function of Na/HCO_3_ exchangers, suggesting that cytosolic Cl^−^ has previously unrecognized regulatory functions (Shcheynikov et al., 2015).

### Collapsing Organellar Membrane Potentials

We have previously shown that under normal Cl^−^ conditions, both bafilomycin and FCCP reduce the NO-dependent shift in E_GABA_ (McMains and Gleason, 2011) but do not eliminate it. With acute bafilomycin exposure in zero Cl^−^ conditions we see a reduction in the NO-dependent release of Cl^−^. The rapid nature of this effect implies that the AOs involved have a substantial proton leak. Here, we also demonstrate that with the store isolated from all other sources of Cl^−^, FCCP completely inhibits the release of Cl^−^. This difference in the effect of FCCP in the presence and absence of external and cytosolic Cl^−^ may be a reflection of the normal influence that extracellular Cl^−^ and plasma membrane Cl^−^ transporters have on both cytosolic and internal Cl^−^. Because of the well documented effects of FCCP in collapsing the mitochondrial membrane potential (Buckler and Vaughan-Jones, 1998; Nicholls, 2006), the action of FCCP also raises the possibility that mitochondria contribute to the NO-dependent release of internal Cl^−^. Garcia et al. (1997) provide evidence for a mitochondrial Cl^−^ store in pituitary lactotrophs that contributes to relatively high levels of cytosolic Cl^−^ found in those cells. Cl^−^ channel currents have also been detected directly in the inner mitochondrial membrane but they appear to have diverse properties and for the most part, their molecular identity is unknown (for review, see Tomaskova and Ondrias, 2010). However, MA and CQ should have no effect on mitochondria because the mitochondrial matrix is basic.

### Increasing AO pH

We have previously shown that acidification of the cytosol by inhibition of the NHE shifts E_GABA_ positively and alkalization promotes a negative shift in E_GABA_ indicating an increase and decrease in cytosolic Cl^−^, respectively (McMains and Gleason, 2011). With internal MA and CQ, deprotonation before entry into AOs, will have some acidifying effect on the cytosol that will be mitigated by plasma membrane NHE activity and the 10 mM HEPES in the pipet. Thus, any acidification of the cytosol is likely to be more transient than the increase in AO pH. Although the cytosolic acidification could contribute to the increase in cytosolic Cl^−^ observed in MA and CQ the contribution should be relatively small because inhibition of NHE with amiloride generated an E_GABA_ shift of only +5 mV whereas MA and CQ shifted E_GABA_ by +18 mV and +14 mV, respectively. It is also important to note that our results in normal internal and external Cl^−^ were consistent with GABA pulse experiments where the only source of Cl^−^ was compartmental. With MA and CQ, AO pH is raised but the membrane potential is intact. This raises the question of how Cl^−^ can move out of AO against an inward-positive membrane potential. Under zero cytosolic Cl^−^ conditions, the AO Vm could be substantial (Van Dyke, 1988; Grabe and Oster, 2001) but the driving force on Cl^−^ would still be outward, at least up to a point. Considering our results in normal cytosolic Cl^−^, the picture is more complicated because the driving force on Cl^−^ will be smaller. However, an additional consideration is that the AO Vm could be compensated by cation flux (Steinberg et al., 2010). Clearly, there is much to be learned about the physiology and functions of ion flux across AO membranes (Scott and Gruenberg, 2011). While there are multiple mechanisms available to move Cl^−^, these experiments reveal that compartmental Cl^−^ transport mechanisms respond to changes in pH within the compartment itself as well as pH changes in the cytosol.

The effects of cytosolic and AO pH changes on basal cytosolic Cl^−^ levels are, however, quite distinct from the effects of those factors on the NO-dependent release of Cl^−^. In our previous work, we discovered that alkalization of the cytosol suppressed the NO-dependent shift in E_GABA_ while acidification had no significant effect on the NO-dependent shift (McMains and Gleason, 2011). Here, we see that raising AO pH actually reverses the effect of NO such that E_GABA_ is shifted in the negative direction consistent with removal of Cl^−^ from the cytosol. The mechanisms of this reversal is not yet understood. One of our working hypotheses is that internal ClC H/Cl exchangers mediate the NO-dependent release of compartmental Cl^−^ that we observe under control conditions. Indeed, we have RT-PCR and immunocytochemical evidence that ClCs 3, 4, 5, 6 and 7 are expressed in amacrine cells (McMains et al., 2011). Outward movement of Cl^−^ and inward movement of H^+^ conform to the preferred direction of transport demonstrated by current measurements from ClCs 3, 4 and 5 expressed in heterologous systems (Friedrich et al., 1999; Li et al., 2000; Scheel et al., 2005; Smith and Lippiat, 2010). The increase in cytosolic Cl^−^ we see upon elevation of compartmental pH with MA and CQ would be consistent with ClC involvement because the coupled import of H^+^ and export of Cl^−^ is favored under these conditions, at least for ClC5 (Smith and Lippiat, 2010). However, the resistance of the ClC exchangers to reversal makes it unlikely that these transporters could mediate NO-dependent Cl^−^ uptake. Instead, we are compelled to envision either a complex interaction between compartmental transport mechanisms or activation of Cl^−^ export at the plasma membrane that is promoted by an increase in compartmental pH plus NO.

### Potential Sources of NO-releasable Cl^−^

AOs include the Golgi, endosomes, synaptic vesicles, and lysosomes. The Golgi complex is typically located in neuronal cell bodies but Golgi outposts have been discovered that assemble in dendrites (Gardiol et al., 1999; Horton et al., 2005) and even in dendritic spines (Pierce et al., 2001). Thus in neurons, the distribution of Golgi can be nearly as widespread as endosomes and lysosomes. The Golgi is not as acidic as endosomes and lysosomes but all three contain Cl^−^ and Cl^−^ transport proteins including ClCs (Edwards and Kahl, 2010). We are actively pursuing the identification of the Cl^−^ transporter that mediates the NO-dependent release of internal Cl^−^. Once in hand, this information will allow us use co-localization studies with AO-specific markers to assess the relative contributions of these three classes of AO.

Because establishment of proton gradients can consume ATP (AOs) or are required to generate ATP (mitochondria), the movement of Cl^−^ is linked not only to protons but also to the energetic balance of the cell and its organelles. In cells with complex architectures such as neurons, cytosolic Cl^−^ levels also have the potential to be spatially diverse. Amacrine cells have complex dendritic morphology and local synaptic interactions dominate (Marc and Liu, 2000; Marc et al., 2014). The possibility that synaptic vesicles can be a source of Cl^−^ is intriguing, especially in amacrine cells because these cells participate in reciprocal synapses where pre- and postsynaptic sites can be adjacent. Thus, it is important to understand how postsynaptic cytosolic Cl^−^ levels are regulated and ultimately, how highly localized regulation might occur. Our results thus far suggest that local cytosolic Cl^−^ levels might be influenced by regional cytosolic and organellar pH changes as well as synapse-specific generation of NO.

## Author Contributions

VK contributed to the design of the experiments and executed all of the experiments. VK also contributed to the drafting and revising of the text and figures and approved of the final version of this manuscript. EG contributed to the design of the experiments. EG also contributed to the drafting and revising of the text and figures and approved of the final version of this manuscript.

## Conflict of Interest Statement

The authors declare that the research was conducted in the absence of any commercial or financial relationships that could be construed as a potential conflict of interest.

## References

[B1] Abreu B. J. Guimarães M. Uliana L. C. Vigh J. von Gersdorff H. Prado M. A.. (2008). Protein kinase C modulates synaptic vesicle acidification in a ribbon type nerve terminal in the retina. Neurochem. Int. 53, 155–164. 10.1016/j.neuint.2008.07.004 18691623 PMC3612021

[B2] Bormann J. Hamill O. P. Sakmann B. (1987). Mechanism of anion permeation through channels gated by glycine and gamma-aminobutyric acid in mouse cultured spinal neurones. J. Physiol. 385, 243–286. 10.1113/jphysiol.1987.sp016493 2443667 PMC1192346

[B3] Buckler K. J. Vaughan-Jones R. D. (1998). Effects of mitochondrial uncouplers on intracellular calcium, pH and membrane potential in rat carotid body type I cells. J. Physiol. 513(Pt. 3), 819–833. 10.1111/j.1469-7793.1998.819ba.x 9824720 PMC2231310

[B4] Cousin M. A. Nicholls D. G. (1997). Synaptic vesicle recycling in cultured cerebellar granule cells: role of vesicular acidification and refilling. J. Neurochem. 69, 1927–1935. 10.1046/j.1471-4159.1997.69051927.x 9349537

[B5] Edwards J. C. Kahl C. R. (2010). Chloride channels of intracellular membranes. FEBS Lett. 584, 2102–2111. 10.1016/j.febslet.2010.01.037 20100480 PMC2929963

[B6] Fiedler T. J. Davey C. A. Fenna R. E. (2000). X-ray crystal structure and characterization of halide-binding sites of human myeloperoxidase at 1.8 A resolution. J. Biol. Chem. 275, 11964–11971. 10.1074/jbc.275.16.11964 10766826

[B7] Forgac M. Cantley L. Wiedenmann B. Altstiel L. Branton D. (1983). Clathrin-coated vesicles contain an ATP-dependent proton pump. Proc. Natl. Acad. Sci. U S A 80, 1300–1303. 10.1073/pnas.80.5.1300 6131417 PMC393584

[B8] Friedrich T. Breiderhoff T. Jentsch T. J. (1999). Mutational analysis demonstrates that ClC-4 and ClC-5 directly mediate plasma membrane currents. J. Biol. Chem. 274, 896–902. 10.1074/jbc.274.2.896 9873029

[B9] Fuchs R. Mâle P. Mellman I. (1989). Acidification and ion permeabilities of highly purified rat liver endosomes. J. Biol. Chem. 264, 2212–2220. 2914902

[B10] Garcia L. Rigoulet M. Georgescauld D. Dufy B. Sartor P. (1997). Regulation of intracellular chloride concentration in rat lactotrophs: possible role of mitochondria. FEBS Lett. 400, 113–118. 10.1016/s0014-5793(96)01365-8 9000524

[B11] Gardiol A. Racca C. Triller A. (1999). Dendritic and postsynaptic protein synthetic machinery. J. Neurosci. 19, 168–179. 9870948 10.1523/JNEUROSCI.19-01-00168.1999 PMC6782360

[B12] Gleason E. Borges S. Wilson M. (1993). Synaptic transmission between pairs of retinal amacrine cells in culture. J. Neurosci. 13, 2359–2370. 8099124 10.1523/JNEUROSCI.13-06-02359.1993 PMC6576475

[B13] Glykys J. Dzhala V. Egawa K. Balena T. Saponjian Y. Kuchibhotla K. V.. (2014). Local impermeant anions establish the neuronal chloride concentration. Science 343, 670–675. 10.1126/science.1245423 24503855 PMC4220679

[B14] Grabe M. Oster G. (2001). Regulation of organelle acidity. J. Gen. Physiol. 117, 329–344. 10.1085/jgp.117.4.329 11279253 PMC2217256

[B15] Hentschke M. Wiemann M. Hentschke S. Kurth I. Hermans-Borgmeyer I. Seidenbecher T.. (2006). Mice with a targeted disruption of the Cl−/HCO3- exchanger AE3 display a reduced seizure threshold. Mol. Cell. Biol. 26, 182–191. 10.1128/mcb.26.1.182-191.2006 16354689 PMC1317631

[B16] Hilgen G. Huebner A. K. Tanimoto N. Sothilingam V. Seide C. Garrido M. G.. (2012). Lack of the sodium-driven chloride bicarbonate exchanger NCBE impairs visual function in the mouse retina. PLoS One 7:e46155. 10.1371/journal.pone.0046155 23056253 PMC3467262

[B51] Hoffpauir B. Gleason E. (2002). Activation of mGluR5 modulates GABAA receptor function in retinal amacrine cells. J. Neurophysiol. 88, 1766–1776. 10.1152/jn.00174.2002 12364505

[B17] Hoffpauir B. McMains E. Gleason E. (2006). Nitric oxide transiently converts synaptic inhibition to excitation in retinal amacrine cells. J. Neurophysiol. 95, 2866–2877. 10.1152/jn.01317.2005 16467419

[B18] Hörnberg A. Hultdin U. W. Olofsson A. Sauer-Eriksson A. E. (2005). The effect of iodide and chloride on transthyretin structure and stability. Biochemistry 44, 9290–9299. 10.1021/bi050249z 15981995

[B19] Horton A. C. Rácz B. Monson E. E. Lin A. L. Weinberg R. J. Ehlers M. D. (2005). Polarized secretory trafficking directs cargo for asymmetric dendrite growth and morphogenesis. Neuron 48, 757–771. 10.1016/j.neuron.2005.11.005 16337914

[B20] Jacobs S. Ruusuvuori E. Sipilá S. T. Haapanen A. Damkier H. H. Kurth I.. (2008). Mice with targeted Slc4a10 gene disruption have small brain ventricles and show reduced neuronal excitability. Proc. Natl. Acad. Sci. U S A 105, 311–316. 10.1073/pnas.0705487105 18165320 PMC2224208

[B21] Kaila K. Price T. J. Payne J. A. Puskarjov M. Voipio J. (2014). Cation-chloride cotransporters in neuronal development, plasticity and disease. Nat. Rev. Neurosci. 15, 637–654. 10.1038/nrn3819 25234263 PMC4294553

[B22] Kuner T. Augustine G. J. (2000). A genetically encoded ratiometric indicator for chloride: capturing chloride transients in cultured hippocampal neurons. Neuron 27, 447–459. 10.3410/f.1001096.17167 11055428

[B23] Li X. Shimada K. Showalter L. A. Weinman S. A. (2000). Biophysical properties of ClC-3 differentiate it from swelling-activated chloride channels in Chinese hamster ovary-K1 cells. J. Biol. Chem. 275, 35994–35998. 10.1074/jbc.m002712200 10973952

[B24] Linser P. Moscona A. A. (1984). Variable CA II compartmentalization in vertebrate retina. Ann. N Y Acad. Sci. 429, 430–446. 10.1111/j.1749-6632.1984.tb12369.x 6430181

[B25] Marc R. E. Anderson J. R. Jones B. W. Sigulinsky C. L. Lauritzen J. S. (2014). The AII amacrine cell connectome: a dense network hub. Front. Neural Circuits 8:104. 10.3389/fncir.2014.00104 25237297 PMC4154443

[B26] Marc R. E. Liu W. (2000). Fundamental GABAergic amacrine cell circuitries in the retina: nested feedback, concatenated inhibition and axosomatic synapses. J. Comp. Neurol. 425, 560–582. 10.1002/1096-9861(20001002)425:4<560::aid-cne7>3.0.co;2-d 10975880

[B27] Masland R. H. (2012). The neuronal organization of the retina. Neuron 76, 266–280. 10.1016/j.neuron.2012.10.002 23083731 PMC3714606

[B28] McMains E. Gleason E. (2011). Role of pH in a nitric oxide-dependent increase in cytosolic Cl(-) in retinal amacrine cells. J. Neurophysiol. 106, 641–651. 10.1152/jn.00057.2011 21593387 PMC3154806

[B29] McMains E. Krishnan V. Prasad S. Gleason E. (2011). Expression and localization of CLC chloride transport proteins in the avian retina. PLoS One 6:e17647. 10.1371/journal.pone.0017647 21408174 PMC3049779

[B30] Nicholls D. G. (2006). Simultaneous monitoring of ionophore- and inhibitor-mediated plasma and mitochondrial membrane potential changes in cultured neurons. J. Biol. Chem. 281, 14864–14874. 10.1074/jbc.m510916200 16551630

[B31] Ohkuma S. Poole B. (1978). Fluorescence probe measurement of the intralysosomal pH in living cells and the perturbation of pH by various agents. Proc. Natl. Acad. Sci. U S A 75, 3327–3331. 10.1073/pnas.75.7.3327 28524 PMC392768

[B32] Payne J. A. Rivera C. Voipio J. Kaila K. (2003). Cation-chloride co-transporters in neuronal communication, development and trauma. Trends Neurosci. 26, 199–206. 10.1016/s0166-2236(03)00068-7 12689771

[B33] Pierce J. P. Mayer T. McCarthy J. B. (2001). Evidence for a satellite secretory pathway in neuronal dendritic spines. Curr. Biol. 11, 351–355. 10.1016/s0960-9822(01)00077-x 11267872

[B34] Piwon N. Günther W. Schwake M. Bösl M. R. Jentsch T. J. (2000). ClC-5 Cl− channel disruption impairs endocytosis in a mouse model for Dent’s disease. Nature 408, 369–373. 10.1038/35042597 11099045

[B35] Poole B. Ohkuma S. (1981). Effect of weak bases on the intralysosomal pH in mouse peritoneal macrophages. J. Cell Biol. 90, 665–669. 10.1083/jcb.90.3.665 6169733 PMC2111912

[B36] Rybak S. L. Lanni F. Murphy R. F. (1997). Theoretical considerations on the role of membrane potential in the regulation of endosomal pH. Biophys. J. 73, 674–687. 10.1016/s0006-3495(97)78102-5 9251786 PMC1180966

[B37] Scheel O. Zdebik A. A. Lourdel S. Jentsch T. J. (2005). Voltage-dependent electrogenic chloride/proton exchange by endosomal CLC proteins. Nature 436, 424–427. 10.1038/nature03860 16034422

[B38] Scott C. C. Gruenberg J. (2011). Ion flux and the function of endosomes and lysosomes: pH is just the start: the flux of ions across endosomal membranes influences endosome function not only through regulation of the luminal pH. Bioessays 33, 103–110. 10.1002/bies.201000108 21140470

[B39] Shcheynikov N. Son A. Hong J. H. Yamazaki O. Ohana E. Kurtz I.. (2015). Intracellular Cl− as a signaling ion that potently regulates Na+/HCO3- transporters. Proc. Natl. Acad. Sci. U S A 112, E329–E337. 10.1073/pnas.1415673112 25561556 PMC4311818

[B40] Smith A. J. Lippiat J. D. (2010). Direct endosomal acidification by the outwardly rectifying CLC-5 Cl(-)/H(+) exchanger. J. Physiol. 588, 2033–2045. 10.1113/jphysiol.2010.188540 20421284 PMC2911210

[B41] Sonawane N. D. Verkman A. S. (2003). Determinants of [Cl−] in recycling and late endosomes and Golgi complex measured using fluorescent ligands. J. Cell Biol. 160, 1129–1138. 10.1083/jcb.200211098 12668661 PMC2172765

[B42] Stauber T. Jentsch T. J. (2013). Chloride in vesicular trafficking and function. Annu. Rev. Physiol. 75, 453–477. 10.1146/annurev-physiol-030212-183702 23092411

[B43] Steinberg B. E. Huynh K. K. Brodovitch A. Jabs S. Stauber T. Jentsch T. J.. (2010). A cation counterflux supports lysosomal acidification. J. Cell Biol. 189, 1171–1186. 10.1083/jcb.200911083 20566682 PMC2894458

[B44] Tomaskova Z. Ondrias K. (2010). Mitochondrial chloride channels–What are they for? FEBS Lett. 584, 2085–2092. 10.1016/j.febslet.2010.01.035 20100478

[B45] Van Dyke R. W. (1988). Proton pump-generated electrochemical gradients in rat liver multivesicular bodies. Quantitation and effects of chloride. J. Biol. Chem. 263, 2603–2611. 2963813

[B46] Venkataramani S. Van Wyk M. Buldyrev I. Sivyer B. Vaney D. I. Taylor W. R. (2014). Distinct roles for inhibition in spatial and temporal tuning of local edge detectors in the rabbit retina. PLoS One 9:e88560. 10.1371/journal.pone.0088560 24586343 PMC3931627

[B47] Wang S. S. Devuyst O. Courtoy P. J. Wang X. T. Wang H. Wang Y.. (2000). Mice lacking renal chloride channel, CLC-5, are a model for Dent’s disease, a nephrolithiasis disorder associated with defective receptor-mediated endocytosis. Hum. Mol. Genet. 9, 2937–2945. 10.1093/hmg/9.20.2937 11115837

[B48] Wu M. M. Llopis J. Adams S. McCaffery J. M. Kulomaa M. S. Machen T. E.. (2000). Organelle pH studies using targeted avidin and fluorescein-biotin. Chem. Biol. 7, 197–209. 10.1016/s1074-5521(00)00088-0 10712929

[B49] Zhou Z. J. Lee S. (2008). Synaptic physiology of direction selectivity in the retina. J. Physiol. 586, 4371–4376. 10.1113/jphysiol.2008.159020 18617561 PMC2614022

[B50] Zhou P. Tian F. Zou J. Ren Y. Liu X. Shang Z. (2010). Do halide motifs stabilize protein architecture? J. Phys. Chem. B 114, 15673–15686. 10.1021/jp105259d 21049982

